# Factors linked to virological failure in people on a dolutegravir-based regimen in Mamelodi

**DOI:** 10.4102/sajid.v39i1.670

**Published:** 2024-10-04

**Authors:** Moloko S. Mmatsoku, Sanele Ngcobo

**Affiliations:** 1 School of Health Systems and Public Health, Faculty of Health Sciences, University of Pretoria, Pretoria, South Africa; 2 Department of Family Medicine, Faculty of Health Sciences, University of Pretoria, Pretoria, South Africa

**Keywords:** dolutegravir, antiretroviral therapy, HIV, viral load, tenofovir disoproxil fumarate lamivudine, factors

## Abstract

**Background:**

Since 2019, the World Health Organization has recommended dolutegravir-containing regimens for HIV in low- and middle-income countries because of its high genetic barriers to resistance, lower drug interactions, fewer side effects, higher viral load (VL) suppression rates and cost-effectiveness compared to efavirenz.

**Objectives:**

This study investigates factors associated with unsuppressed VLs in people living with HIV on tenofovir-lamivudine and dolutegravir (TLD) in South Africa (SA).

**Method:**

A cross-sectional study was conducted between October 2023 and February 2024 at Mamelodi Regional Hospital’s Ntshembo Clinic. Participants were people living with HIV aged 18 years and older, more than 6 months on TLD, with either suppressed (≤ 50 copies/mL) or unsuppressed (> 50 copies/mL) VLs.

**Results:**

Significant associations were found between unsuppressed VL and factors such as sex, marital status, occupation and education level. Male participants were less likely to achieve VL suppression than female participants (odds ratio: 0.45, *p* = 0.0007). Poor antiretroviral therapy adherence was linked to higher unsuppressed VL (*p* < 0.05). Newly initiated patients had significantly lower suppression rates (*p* < 0.05). The use of traditional or herbal and religious products was also linked to unsuppressed VL (*p* < 0.05).

**Conclusion:**

The study highlights the importance of addressing adherence factors to improve VL suppression rates among people living with HIV on TLD.

**Contribution:**

Tailored interventions targeting adherence, especially among newly initiated patients, and addressing the use of traditional or herbal and religious products are warranted to enhance treatment outcomes.

## Introduction

Since 2019, the World Health Organization (WHO) has recommended that low- and middle-income countries use dolutegravir (DTG) containing regimens in first- and second-line antiretroviral therapy (ART) for HIV.^1,2^ The DTG is an integrase strand transfer inhibitor (InSTI) a high genetic barrier to resistance, few drug interactions, no interaction with contraceptives, fewer side effects, high rate of viral load (VL) suppression and cost-effectiveness compared to efavirenz (EFV).^3^

The currently preferred fixed-dose combination (FDC) DTG-containing regimen is tenofovir, lamivudine (3TC), and DTG, commonly known as tenofovir-lamivudine and dolutegravir (TLD), which is replacing TDF, emtricitabine (FTC) and EFV, known as TEE.^3,4^

In 2007 and 2022, Statistics South Africa reported 278 741 and 85 796 AIDS related deaths, respectively.^5,6^ The decline of 192 945 deaths was because of greater access to ART, which was well tolerated and had good adherence.^5^ Optimal ART involves the best cost-effective and efficient medications.^7^

The primary purpose of ART is to suppress the HIV VL copies; the South African HIV Guidelines 2019 defined it as VL ≤ 50 copies per mL.^3^ The suppressed VL leads to a restored and preserved immune system and improves the health of people living with HIV while increasing their life span.^4,8^ Furthermore, suppressive ART reduces perinatal HIV transmission.^4,8^ People living with HIV cannot sexually transmit HIV to their HIV-negative sexual partners, endorsing the ‘Undetectable equals Untransmittable’ campaign (U = U).^4,8^

The Southern African HIV Clinician Society (SAHCS) May 2022 publication advised that people living with HIV on TEE can be switched to TLD regardless of the VL.^9^ Southern African HIV Clinician Society further advised that people with a suppressed VL within the last 6 months on the protease inhibitor (PI) regimen such as lopinavir and ritonavir (LPV/r) or atazanavir/ritonavir (ATV/r) can be switched to TLD regardless of any resistance pattern or history of the treatment.^9^

The 2023 National Department of Health ART clinical guidelines endorsed the SAHCS recommendations.^9,10^ The achievement of The Joint United Nations Programme on HIV and AIDS (UNAIDS) third 95 means that 95% of people living with HIV on the optimised ART, which is TLD currently, must obtain a suppressed VL (≤ 50 c/mL); this is very critical to the concept of U = U.^8,11^ This subsequently results in the decline of sexually transmitted and perinatal new HIV infections to end the AIDS epidemic. The UNAIDS aims to end the epidemic in 2030, achievable through TLD adherence.^8,11^

There have been few studies relating to factors associated with unsuppressed HIV VL on TLD. A Malawian prospective cohort study stated in its interpretation that within a year of the introduction of transitioning patients to TLD, a high VL suppression was noticed.^12^ Of the 1892 participants in a Malawian study, 1725 (97.9%) (confidence intervals [CI]: 97.1–98.5) of 1762 reported a VL of > 51 c/mL. This study highlighted adherence challenges but without detail.^12^ They recommended resistance and HIV VL surveillance during this massive transition to TLD.^12^

A retrospective study from Brazil between 2014 and 2017 used programmatic HIV data to assess the effectiveness of the first-line ART regimen in achieving VL suppression over the first 12 months.^13^ TDF, 3TC and DTG at 90.5% (95% CI 90.0–91.0) were more effective than TDF, 3TC and EFV at 84.0% (95% CI 83.7–84.2).^13^ The data showed that DTG was superior to the EFV and protease-inhibitor-based regimens for VL suppression; however, factors associated with VL suppression were not reported.^13^

A cross-sectional study on antiretroviral (ARV) non-adherence was conducted in a Kenyan referral hospital (Turkana County).^14^ A lack of education, support or food, distance to the health facility, side effects and alcohol usage were associated with non-adherence. Of the 5243 people living with HIV who had initiated ART; only 1551 (30%) were on treatment, a 70% reduction.^14^ This study investigated and identified factors associated with unsuppressed VL in people living with HIV on TLD in a South African clinic.

## Research methods and design

The study design was a cross-sectional study, utilising a questionnaire to collect data from patients on a TLD-containing regimen.

The study was conducted at Mamelodi Regional Hospital in the Tshwane district, approximately 12.7 km from South Africa’s capital city, Tshwane. The hospital, under Region Six, serves residents of Mamelodi, Pretoria East, Eersterust and Nellmapius. Newly diagnosed HIV patients from the wards and specialised outpatient departments are referred to the Ntshembo Clinic, which specialises in HIV and AIDS and Tuberculosis care. Patients may either continue their care at Ntshembo or be transferred to nearby local health facilities.

The study participants were primarily people living with HIV receiving treatment at the Ntshembo Clinic, focusing on those on a TLD regimen. Selection criteria included being aged 18 years and older who had been on a TLD regimen for over 6 months, categorised based on their latest VL results obtained within the last 6 months from the questionnaire date: VL > 50 c/mL or VL ≤ 50 c/mL. Exclusion criteria encompassed people living with HIV younger than 18, individuals not on a TLD regimen or those on TLD for less than 6 months. The principal investigator conducted participant selection over 3 months at Mamelodi Regional Hospital’s Ntshembo Clinic, following standardised recruitment guidelines. During regular clinic hours from 07h00 to 08h00, the investigator retrieved patient files with assistance from Mamelodi Hospital data capture. Participants underwent the clinic routine, including accessing their files, attending counselling sessions and visiting the vital signs room for measurements. The clinic utilises Tier.net (an electronic record system for managing HIV/AIDS care), managing 1179 people living with HIV on TLD, with 785 having suppressed and 394 having unsuppressed VLs. Recruitment included participants with clinic appointments between 30 October 2023 and 28 February 2024. All recruited participants received detailed study briefings and voluntarily signed informed consent documents. The principal investigator administered an online questionnaire during clinic visits, taking approximately 10 min and supplemented data with information from participants’ clinic files.

### Data sources and measurement

The researcher developed the questionnaire using literature and existing validated instruments, including the Adherence Barriers Questionnaire for HIV (ABQ-HIV) patients on ART and the Alcohol, Smoking and Substance Involvement Screening Test (ASSIST). Once designed, the questionnaire was entered into Qualtrics Online Survey, University of Pretoria version, for data collection purposes. To test the feasibility of the study and the questionnaire, a pilot study was conducted on five participants; data from this pilot study did not form part of the primary study. The questionnaire covered biographic information, HIV status disclosure, patient support systems, details about TLD treatment, adherence, substance use and medical history.

### Sample size

Study participants were purposefully sampled based on their VL results. A sample size of 290 study participants was calculated using an online sample size calculator, considering the assumed population size of 1179 people living with HIV on TLD at Mamelodi Hospital. This calculation factored in clinic appointments between 30 October 2023 and 28 February 2024, with a margin of error of 5% for 95% CI and a 50% prevalence to ensure maximum sample size.

### Statistical analysis

The data analysis was conducted to assess factors associated with VL suppression among the 304 study participants recruited from Mamelodi Hospital between 30 October 2023 and 28 February 2024. Descriptive statistics were used to summarise participant demographics, including sex, marital status, occupational status and education level. The Chi-square test was employed to examine associations between categorical variables such as sex, marital status and VL suppression status, reporting odds ratios (OR) with 95% CI and corresponding *p*-values.

In addition, logistic regression analysis was performed to further explore the relationships between demographic factors sex, marital status, occupation, education), HIV-related factors (HIV status disclosure, support in ARV adherence) and behavioural factors (medication adherence, health perception) with VL suppression status. For Likert scale responses assessing medication adherence and health perception, independent samples *t*-tests were used to compare mean scores between participants with suppressed and unsuppressed VL. All statistical analyses were conducted using SPSS version 27.0 (IBM Corp., Armonk, NY, USA), with statistical significance set at *p* < 0.05.

### Ethical considerations

The University of Pretoria Faculty of Health Science Research Ethics Committee reviewed the research proposal and provided ethical approval (Ethics reference number: 453/2023). Participants were allocated study numbers e.g. A001 to A304, which were aligned with the clinic file; however, this was only known to the study principal researcher and the study supervisor. The 304 recruited participants were briefed about the study and voluntarily signed an informed consent document that they agreed to participate.

## Results

Of the 304 participants recruited, 41.1% (*n* = 146) of male participants had a suppressed VL, versus 60.8% (*n* = 158) of female participants (OR: 0.45 [95% CI: 0.28 to 0.7]) signifying a 55% lower likelihood of achieving VL suppression among male participants than female participants. Married (48.1%, OR: 0.52, 95% CI: 0.31–0.85, *p* = 0.018) and widowed (12.5%, OR: 0.08, 95% CI: 0.01–0.67, *p* = 0.0163) participants demonstrated significantly lower suppression rates than those who were single (53.8%). In terms of occupational status, self-employed participants (40.0%, OR: 0.35, 95% CI: 0.13–0.93, *p* = 0.0355) and employed participants (51.6%, OR: 0.56, 95% CI: 0.35–0.90, *p* = 0.0163) exhibited significantly lower rates of VL suppression than unemployed participants (53.1%). Participants who reported completing secondary education as their highest level of education had a significantly lower proportion of suppressed VL (45.0%, OR: 0.60, 95% CI: 0.37–0.96, *p* = 0.0335) when compared to those who completed primary (42.2%) (Table 1).

**TABLE 1:** Characteristics of study participants.

Characteristic	Total		Suppressed VL		Unsuppressed VL		OR	95% CI	*p*
	*n*	%	*n*	%	*n*	%			
**Gender**									
Male	146	42.9	60	41.1	86	58.9	0.45	0.28–0.71	0.0007*
Female	158	57.1	96	60.8	62	39.2	Ref	-	-
**Marital status**									
Single	186	61.2	100	53.8	86	46.2	Ref	-	-
Married	104	34.2	50	48.1	54	51.9	0.52	0.31–0.85	0.0108
Divorced	6	2.0	5	83.3	1	16.7	2.80	3.20–24.50	0.3500
Widowed	8	2.6	1	12.5	7	87.5	0.08	0.01–0.67	0.0196
**Occupation**									
Unemployed	81	26.7	43	53.1	38	46.9	Ref	-	-
Self-employed	20	6.6	8	40.0	12	60.0	0.35	0.13–0.93	0.0355
Employed	192	63.4	99	51.6	93	48.4	0.56	0.35–0.90	0.0163
Student	9	3.0	4	44.4	5	55.6	0.42	0.11–1.66	0.2191
Other	1	0.3	1	100.0	0	0.0	1.60	0.06–40.15	0.7746
**Highest level of education**									
Never went to school	15	4.9	10	66.7	5	33.3	1.46	0.47–4.52	0.5118
Completed primary school	128	42.1	74	57.8	54	42.2	Ref	-	-
Completed secondary school	149	49.0	67	45.0	82	55.0	0.60	0.37–0.96	0.0335
Completed tertiary education	12	4.0	5	41.7	7	58.3	0.52	0.16–1.73	0.2873

CI, confidence interval; OR, odds ratio; Ref, reference; VL, viral load.

* , Statistically significant *p*-values.

### HIV status disclosure and support

Of the 297 participants who indicated that they disclosed their HIV status, 52.2% (*n* = 155) exhibited a suppressed VL compared to 14.3% (*n* = 1) among those who did not disclose their status (*n* = 7). Among 179 participants who reported receiving support in taking ARVs, 52% had suppressed VL, compared to 45.8% of those who reported not getting support. Notably, those getting support from people living with HIV had the highest suppression rate of 53.9%, followed by those supported by HIV-negative individuals (51.5%) (Table 2). The analysis did not reveal any statistically significant disparities in the levels of disclosure and support pertaining to both VL suppression and unsuppression.

**TABLE 2:** HIV status disclosure and support.

Variable	Total		Suppressed VL		Unsuppressed VL		OR	95% CI	*p*
	*n*	%	*n*	%	*n*	%			
**HIV status disclosure**									
No	7	2.3	1	14.3	6	85.7	Ref	-	Ref
Yes	297	97.7	155	52.2	142	47.8	6.51	0.77–54.72	0.0847
**Support for taking ARVs**									
No	24	7.9	11	45.8	13	54.2	Ref	-	Ref
Yes	279	92.1	145	52.0	134	48.0	1.28	0.55–2.95	0.5600
**Supporter’s known HIV status**									
Yes	254	91.0	130	51.2	124	48.8	Ref	-	-
No	25	9.0	15	60.0	10	40.0	1.43	0.62–3.30	0.5645
**HIV status of support person**									
HIV positive	152	59.8	82	53.9	70	46.1	Ref	-	-
HIV negative	68	26.8	35	51.5	33	48.5	0.91	0.51–1.61	0.7337
Prefer not to answer	34	13.4	13	38.2	21	61.8	0.53	0.25–1.13	0.1008

ARV, antiretroviral; CI, confidence interval; OR, odds ratio; Ref, reference; VL, viral load.

### Patient perspective on their medication adherence and health perception

A grading system ranging from ‘1’ (indicating strong disagreement) to ‘4’ (strong agreement) was used to measure patients’ perspectives on their medication adherence. The mean Likert scale score for displaying higher trust in a clinician was significantly greater in the VL-suppressed group (mean: 3.91, standard deviation [s.d.]: 0.37) than in the unsuppressed group (mean: 3.70, s.d.: 0.53) (Table 3). Regarding medication effectiveness and strict regularity, the mean was higher in the suppressed VL group (mean: 3.90, s.d.: 0.47) than in the unsuppressed VL group (mean: 3.30, s.d.: 0.77). In the context of ‘taking medicines automatically at a fixed time’, the mean was 3.82 (s.d.: 0.63) for the suppressed group, compared to 3.14 (s.d.: 0.83) for the unsuppressed group, *p* < 0.001. When away from home, adherence challenges to the treatment plan were rated with a mean of 1.30 (s.d.: 0.52) among individuals with suppressed VL, compared to 1.43 (s.d.: 0.73) among those with unsuppressed VL (*p* < 0.001). The family and/or friend support was stronger among suppressed participants, with a mean of 3.88 (s.d.: 0.43) compared to 3.57 (s.d.: 0.73) among those with unsuppressed VL.

**TABLE 3:** Patients’ perspectives on their medication adherence and health perception.

Statements on patient perspectives regarding medication adherence	Suppressed VL		Unsuppressed VL		Total	
	Mean	s.d.	Mean	s.d.	*p*	*t*
I fully understand what my doctor, nurse or pharmacist has explained to me regarding my medication therapy.	4.0	0.1	4.0	0.1	0.207	0.631
I can mention the names of my medicines and their scope without hesitation.	1.2	0.6	1.1	0.4	0.087	0.781
I trust my clinician and agree on my therapy plan together with him.	3.9	0.4	3.7	0.5	< 0.001*	4.003
My medications only help me if I take them on a strict regular basis.	3.9	0.5	3.3	0.8	< 0.001*	8.112
Medicines are all poisonous. You should avoid taking medicines at all if possible.	1.1	0.4	1.1	0.3	0.355	0.464
I feel basically healthy. Therefore, I am sometimes unsure whether I really have to take my medicines daily.	1.4	0.7	1.4	0.8	0.211	−0.421
I take my medicines automatically at a fixed time or on fixed occasions every day (e.g. at meal times, before going to bed).	3.8	0.6	3.1	0.8	< 0.001*	8.050
Generally, I find it unpleasant when other people notice my medication intake.	1.4	0.7	1.3	0.6	0.012	1.545
Generally, I often feel bad, and sometimes I feel discouraged and depressed.	1.3	0.6	1.3	0.6	0.837	0.424
I frequently have problems taking my medications (e.g. swallowing, opening the package, dividing the tablets) or it is difficult for me to adhere to the accompanying conditions of the medication intake (e.g. on an empty stomach, with food or alcohol restrictions).	1.3	0.5	1.4	0.7	0.002	−1.255
I have difficulties adhering to my treatment plan, especially when I am away from home (e.g. at weekends, on business trips or holidays).	1.3	0.5	1.4	0.7	< 0.001*	−1.804
I receive great support from my family members/friends, who I can talk to at any time and ask for help.	3.9	0.4	3.6	0.7	< 0.001*	4.452
In case I have already noticed or in case I were to notice side effects related to my medicines: I have talked or would talk to my doctor about them as soon as possible.	3.3	1.1	3.2	1.1	0.241	1.155
In case I have already noticed or in case I were to notice side effects related to my medicines: I have stopped/would stop taking my medications or would take less of them.	2.1	0.9	2.3	0.8	0.290	−1.936
I frequently forget things on a daily basis.	1.3	0.6	1.3	0.6	0.741	−0.032

*Source:* Mueller S, Wilke T, Gorasso V, Erhart M, Kittner JM. Adaption and validation of the Adherence Barriers Questionnaire for HIV patients on antiretroviral therapy (ABQ-HIV). BMC Infect Dis. 2018;18(1):599. https://doi.org/10.1186/s12879-018-3530-x

VL, viral load; s.d., standard deviation.

* , Statistically significant *p*-values.

The following questionnaire was adapted by the principal investigator using validated questionnaires such as the ABQ-HIV patients on ART.^28^

Regarding adherence in the past 3 months, participants who reported fair (0%, OR: 0.02, 95% CI: 0.00–0.50, *p* = 0.0152), good (0%, OR: 0.01, 95% CI: 0.00–0.11, *p* = 0.0005) and very good (18.4%, OR: 0.11, 95% CI: 0.05–0.26, *p* < 0.0001) adherence exhibited significantly lower rates of VL suppression than those who reported excellent adherence (67.3%). When asked whether they had been taking their medication as prescribed over the past 3 months, participants who answered ‘sometimes’ (4.4%, OR: 0.03, 95% CI: 0.01–0.13, *p* < 0.0001) exhibited a significantly lower VL suppression rate compared to those who answered ‘yes’ (60.2%) (Table 4).

**TABLE 4:** Antiretroviral therapy treatment adherence.

Adherence	Total		Suppressed VL		Unsuppressed VL		OR	95% CI	*p*
	*n*	%	*n*	%	*n*	%			
**ART adherence last month**									
Very poor	2	0.7	1	50.0	1	50.0	0.49	0.03–7.89	0.6119
Fair	8	2.7	0	0.0	8	100.0	0.02	0.00–0.50	0.0152
Good	36	12.1	0	0.0	36	100.0	0.01	0.00–0.11	0.0005*
Very good	38	12.8	7	18.4	31	81.6	0.11	0.05–0.26	< 0.0001*
Excellent	214	71.8	144	67.3	70	32.7	Ref	-	-
**Over the past 3 months, have you been taking your medications regularly as prescribed?**									
Yes	254	83.8	153	60.2	101	39.8	Ref	-	-
Sometimes	45	14.9	2	4.4	43	95.6	0.03	0.01–0.13	< 0.0001*
No	4	1.3	1	25.0	3	75.0	0.22	0.05–2.15	0.1925

ART, antiretroviral therapy; CI, confidence interval; OR, odds ratio; Ref, reference; VL, viral load.

* , Statistically significant *p*-values.

Of the 303 participants, 8.25% (*n* = 25) were diagnosed with another medical condition; among these, 48% (*n* = 12) had a suppressed VL, while 51.4% among those with no other medical condition had a suppressed VL, with no statistical significance. When participants were asked about their work schedule, those who reported working both day and night shifts exhibited a significantly lower VL suppression rate (30%, OR: 0.35, 95% CI: 0.15–0.80, *p* = 0.0126) compared to those working only day shifts (55.4%). In response to the question regarding (how they started taking TLD), 86.51% (*n* = 263) indicated switching to TLD from another regimen. Individuals initiated on TLD exhibited a significantly lower suppression rate of 34.1% (OR: 0.44, 95% CI: 0.22–0.88, *p* = 0.0202) than those who switched from another regimen, with a VL suppression rate of 54.0%. The analysis revealed that VL suppression showed no significant difference in individuals’ responses to questions regarding side effects, nausea and current medication usage. Participants taking traditional medication exhibited a significantly lower VL suppression rate of 11.1% (OR: 0.11, 95% CI: 0.01–0.91, *p* = 0.0409) compared to 52.9% among those not taking traditional medicine. Nine participants who reported taking holy water exhibited a significantly lower suppression rate of zero (OR: 0.05, 95% CI: 0.00–0.82, *p* = 0.0395) compared to 52.9% among the 295 participants who reported not taking holy water (Table 5).

**TABLE 5:** Health characteristics by viral load status.

Variable	Total		Suppressed VL		Unsuppressed VL		OR	95% CI	*p*
	*N*	%	*n*	%	*n*	%			
**Diagnosed with other medical condition?**									
No	278	91.8	143	51.4	135	48.6	Ref	-	-
Yes	25	8.3	12	48.0	13	52.0	0.87	0.38–1.98	0.7419
**Work shift**									
Day shift	177	79.0	98	55.4	79	44.6	Ref	-	-
Night shift	2	0.9	1	50.0	1	50.0	0.81	0.05–13.09	0.8796
Both day and night shift	30	13.4	9	30.0	21	70.0	0.35	0.15–0.80	0.0126
Other	15	6.7	5	33.3	10	66.7	0.40	0.13–1.22	0.1098
**Started taking TLD**									
Switched from another regimen	263	86.5	142	54.0	121	46.0	Ref	-	-
Initiated on TLD	41	13.5	14	34.1	27	65.9	0.44	0.22–0.88	0.0202
**Side effects**									
Yes	78	26.4	41	52.6	37	47.4	1.09	0.65–1.83	0.7504
No	216	73.5	109	50.5	107	49.5	Ref	-	-
**Nausea**									
Yes	43	100.0	18	41.9	25	58.1	0.71	0.36–1.37	0.3042
No	216	73.5	109	50.5	107	49.5	Ref	-	-
**Taking medication for other medical conditions?**									
Yes	17	73.9	7	41.2	10	58.8	Ref	-	-
No	6	26.1	5	83.3	1	16.7	7.14	0.68–75.22	0.1017
**Taking traditional medicine?**									
Yes	9	3.0	1	11.1	8	88.9	0.11	0.01–0.91	0.0409
No	295	97.0	156	52.9	148	50.2	Ref	-	-
**Taking holy water or other products from church?**									
Yes	9	3.0	0	0.0	9	100.0	0.05	0.00–0.82	0.0395
No	295	97.0	156	52.9	148	50.2	Ref	-	-

CI, confidence interval; OR, odds ratio; Ref, reference; TLD, Tenofovir-Lamivudine and Dolutegravir; VL, viral load.

## Discussion

This study investigated and identified factors associated with unsuppressed VL in people living with HIV on TLD for more than 6 months at Mamelodi Hospital. The results revealed that demographic characteristic factors associated with unsuppressed VL were male sex, marital status, occupational status, working both day and night shifts and level of education. Furthermore, other factors included poor ART adherence, newly initiated patients and using traditional or herbal and religious products.

This research revealed that male participants on TLD were less likely to achieve a suppressed VL compared to their female counterparts. This observation aligns with findings from two previous studies conducted in Ethiopia.^16,17^ However, it is worth observing that while these studies focused on ART, they did not specifically target TLD or DTG-based regimens.^16,17^ Furthermore, the significantly lower VL suppression rate among male participants in this study may be attributed to their poorer treatment adherence, as evidenced by fewer male participants reporting excellent adherence (67.6%) than female participants (75.6%). Previous studies have reported that poor treatment outcomes in male participants are linked to poor treatment adherence.^16,17,18,19^ Additionally, the stigmatisation of visiting specialised HIV and AIDS health facilities and cultural masculinity might hinder the VL suppression of male participants who will choose traditional medicine or religious products.^15,20^

It was observed in this study that participants who reported using the traditional medicine concurrently with ART exhibited a VL suppression rate, which was significantly lower at 11.1% compared to those not using at 52.9% (*p* = 0.0409). Notably, the current findings are similar to the study conducted in a Tanzanian tertiary hospital, which reported that patients on ART had increased odds of achieving viral suppression (OR: 1.42, CI: 0.71–2.82) compared to those who took ART and herbal medicinesconcurrently.^21^

The nine participants who started using the holy water concurrently with TLD medication notably demonstrated a reduced suppression rate of zero (OR: 0.05, 95% CI: 0.82, *p* = 0.0395); in contrast to the 52.9% suppression rate reported by 295 participants not using the holy water concurrently with TLD medication.

This study observed that excellent adherence to treatment was associated with significantly higher VL suppression. Impressively, the participants who reported taking the medication regularly as prescribed had a significantly higher suppression rate of 60.2% when compared to those who indicated taking medication sometimes at 4.4% (*p* < 0.0001). This study’s findings are consistent with results from the University of Gondar, Ethiopia, reporting that with good adherence, VL suppression was 260/296 (87.8%). Another Ethiopian study reported that VL suppression was 96.3% among study participants reporting good ART adherence.^22^

Furthermore, associations were observed between unemployment status and VL suppression; the results revealed a significantly lower rate of VL suppression among employed (51.6%) and self-employed (40.0%) compared to unemployed participants (53.1%). Literature on this topic shows mixed results. A systematic review and meta-analysis found that employment generally correlates with better ART adherence, particularly in low- and high-income countries but not in middle-income countries, highlighting the complexity of these factors.^23^ Other studies have reported being employed as a factor contributing to non-adherence.^24,25^ Here, employment negatively impacted medication adherence because of busy schedules, fear of stigmatisation at work, travel and difficulty accessing refills.^24,25^ We did not study-specific employment-related factors leading to poor VL suppression, highlighting the need for a follow-up study to better understand these influences.

This study adds another novel factor in revealing that individuals initiated on TLD exhibited a significantly lower suppression rate of 34.1% (OR: 0.44, 95% CI: 0.22–0.88, *p* = 0.0202) compared to those who transitioned from another ART regimen to a TLD regimen at 54.0%. The observed difference in VL suppression significance between these two groups underscores the paramount importance of treatment adherence. Notably, a higher proportion of participants who switched to TLD from another regimen reported excellent adherence (74.1%) compared to those who were initiated on TLD (56.4%). These results are aligned with the two studies that looked into treatment outcomes of transitioning the people living with HIV from another ART regimen to a TLD-based regimen.^26,27^

There is a need for tailored interventions to address male patients on TLD by possibly establishing sex-specific health services or days to reach better outcomes of VL suppression. Educational and enhanced adherence counselling for traditional medicine and religious products should be used by both counsellors and HIV clinicians during the patient’s visit to the facility. Social development, private donors and non-governmental organisations need to integrate to assist in mitigating the financial burden experienced by unemployed patients. A patient-centered healthcare worker relationship where the patients decide on the ART time and not a decision taken on thier behalf.

This study contributes to the growing evidence that draws attention to the importance of addressing multifaceted psychosocial factors. Ultimately, the findings should lead to improved treatment adherence for HIV management in public health facilities to achieve HIV VL suppression for people living with HIV on the TLD regimen.

The study’s limitations include relying on a questionnaire based on self-reported data, which is subject to recall bias and social desirability bias, potentially affecting the accuracy of the responses. To address this, clinic files were used to verify some of the clinical variables; however, this approach had its own limitation, as some files contained missing data. Stigma was not assessed in this study, which may limit understanding of its impact on viral suppression.

## Conclusion

This cross-sectional study VL in people living with HIV on the TLD regimen. Factors associated with unsuppressed VL included being male, the use of traditional/herbal and religious products, lower educational attainment, non-adherence to medication and a lack of HIV status disclosure. These findings highlight the need for continued psychosocial support services and strategic interventions to address these factors, thereby improving VL suppression outcomes among people living with HIV newly initiated on TLD. In addition, more studies are needed to better understand the impact of traditional/herbal and religious products on VL suppression.
